# Anticipating older populations’ health risk exacerbated by compound disasters based on mortality caused by heart diseases and strokes

**DOI:** 10.1038/s41598-023-43717-3

**Published:** 2023-10-05

**Authors:** Shangde Gao, Yan Wang

**Affiliations:** Department of Urban and Regional Planning and Florida Institute for Built Environment Resilience, University of Florida, Gainesville, FL 32611 USA

**Keywords:** Natural hazards, Risk factors

## Abstract

The health of older populations in the Southeastern U.S. receives threats from recurrent tropical cyclones and extreme heat, which may exacerbate the mortality caused by heart diseases and strokes. Such threats can escalate when these extremes form compound disasters, which may be more frequent under climate change. However, a paucity of empirical evidence exists concerning the health threats of compound disasters, and anticipations regarding the health risks of older populations under future compound disaster scenarios are lacking. Focusing on Florida, which has 67 counties and the second-largest proportion of older populations among U.S. states, we calibrate Poisson regression models to explore older populations’ mortality caused by heart diseases and strokes under single and compound disasters. The models are utilized to estimate the mortality across future disaster scenarios, the changing climate, and the growing population. We identify that under multiple hurricanes or heat, current-month hurricanes or heat can affect mortality more heavily than previous-month hurricanes or heat. Under future scenarios, co-occurring hurricanes and extreme heat can exacerbate the mortality more severely than other disaster scenarios. The same types of compound disasters can coincide with an average of 20.5% higher mortality under RCP8.5-SSP5 than under RCP4.5-SSP2. We assess older populations’ future health risks, alerting health agencies to enhance preparedness for future “worst-case” scenarios of compound disasters and proactively adapt to climate change.

## Introduction

The Southeastern United States has 11.52 million older adults (aged 65 years and older) in 2021, whose health status is confronting severe threats from frequently occurring extremes such as hurricanes and extreme heat events^1,2^. Past studies have found that more than 50% of the deaths caused by Hurricane Katrina and Hurricane Sandy were older populations^2^, and older populations’ visits to emergency departments were significantly higher than those of young adults during the heatwave events^3^. The high-level health risks of the older population in the Southeastern United States are potentially related to the vulnerability of older populations and the hazardous impacts of hurricanes and extreme heat. On the one hand, older populations’ health status can be especially vulnerable to climate and weather extremes^4–6^. Older populations may have pre-existing medical conditions (e.g., diabetes, high blood pressure) and are sensitive to the change of surrounding environment, such as air temperature and air quality^5,7,8^. The extremes (e.g., hurricanes) can also influence the operation of utility facilities and local infrastructure and hinder older populations from maintaining a livable indoor environment or accessing health services in time^4^. In addition, many older adults in the Southeastern United States live isolated. For example, 40.0% of the older populations in Florida live alone in 2021^9^. They can have difficulties in communicating with emergency responders when an emergency occurs^4–7^. On the other hand, hurricanes and extreme heat can affect the health status of older populations with various physical and mental health stressors^10^. Extreme heat can cause heat-related illness and disturb the thermoregulation of human bodies^11^. Hurricanes can cause physical injuries and intensify the stress on human bodies^12^. These extreme events can also cause physical damage to buildings and disrupt vital infrastructure, such as electricity and healthcare facilities, potentially exacerbating older populations' health risks and limiting their access to critical health resources^13–17^.

Hurricanes and extreme heat can especially exacerbate the health risks of older populations regarding heart diseases (ICD-10 codes of I00 to I09, I11, I13, and I20 to I51) and strokes (ICD-10 codes of I60 to I69)^8,18^. Specifically, from 2000 to 2019, the total mortality (number of deaths) caused by heart diseases and strokes among older populations in Florida reached 39,414 in the months when hurricanes occurred (15,397 in the months without hurricanes), and the mortality reached 49,095 in months with extreme heat (14,959 in the months without extreme heat)^18^. During Hurricane Irma in September 2017, heart diseases and strokes were the leading causes of older populations’ emergency department visits in Florida^19^, and 3669 older adults in Florida died due to heart diseases or strokes in September 2017^18^. Similar adverse health consequences were observed in the past extreme heat events. For instance, heatwave was associated with an average daily death among 75 or older populations increase of 28% in Australia from 1988 to 2011^1^. In another case, under the extreme heat in Miami-Dade County, Florida, from June 2019 to July 2019, 1,464 older adults died from heart diseases or strokes^18^.

Under climate change, hurricanes and extreme heat can form *compound disasters*^20–22^, which may further exacerbate older populations’ risks of heart diseases and strokes. Compound disasters can be multiple weather extremes that occur concurrently (co-occurring disaster events) or successively (multi-disaster events), and the impacts of these extremes may be interacted^20–22^. Historical cases of compound disasters of hurricanes and extreme heat have also displayed severe impacts on the mortality caused by heart diseases and strokes among older populations: in the multi-hurricane events of Hurricanes Charley, Frances, Ivan, and Jeanne between August and September of 2004, the mortality caused by heart diseases and strokes among older populations reached 3,930 in the affected counties under the continuous stress of hurricanes^18,23^. In other cases, heatwaves occurred immediately after Hurricane Laura in 2020 and Hurricane Ida in 2021^24,25^. In these cases, hurricanes and extreme heat can have compounding impacts on older populations’ health. For example, the disturbance of vital infrastructure and services caused by hurricanes can hinder older populations from reducing the indoor temperature with household appliances and intensify the heat-induced physical stress on older populations^26^. Especially in the U.S., older populations may rely on the sustaining of local unity to maintain indoor environment quality with household appliances during weather extremes, as 61.4% of the survey participants after Hurricane Harvey prepared the air conditioning facilities for mitigating the impacts of hurricanes and protecting themselves from the extreme heat^27^. The future compound disasters of hurricanes and extreme heat may occur frequently and lead to high-level mortality of the older population in the Southeastern U.S., where the older population is increasing rapidly^20–22,28,29^. Knowledge of the potential impacts of compound disasters of hurricanes and extreme heat on older populations is highly demanding for proposing targeted risk mitigation strategies.

However, with the extant studies that investigated older populations’ health risks under single events of hurricanes or extreme heat^14,30–32^, limited work has explored such risks under compound disasters. This may be constrained by the lack of longitudinal datasets, the complex impact mechanism of compound disasters, and the lack of prediction of future disaster scenarios*.* In the research field of the impacts of climate and weather extremes, only a couple of studies have explored the impacts of compound disasters on public health, such as Cherry et al.^33^ and Lowe et al.^34^, but none has focused on older population. Additionally, some studies have analyzed older populations’ health risks and mortality under the impacts of climate change and the exacerbated weather extremes, such as the heatwaves in China, Seoul, Tokyo, and Paris^35–37^. However, these studies mainly concerned sole-event extremes or regarded extremes as isolated, providing insufficient knowledge about older populations’ health risks under compound disasters.

Our central hypothesis is that the impacts of compound disasters can intensify the mortality risk of heart diseases and strokes in older populations, compared to the impacts of singular events. Specifically, the study seeks to answer the following two research questions (RQs):

- *RQ1* Can compound events of hurricanes and extreme heat influence older peoples’ mortality (number of deaths) caused by heart diseases and strokes more severely than sole-event extremes (compound events refer to the multiple hits of similar weather extremes and co-occurrence of hurricanes and extreme heat events)?
- *RQ2* How would the older population’s risk of mortality caused by heart diseases and strokes be exacerbated under future scenarios considering the possible occurrence of compound disasters of hurricanes and extreme heat?

To answer the research questions, we focus on the state of Florida, U.S., which has the second-highest proportion of older population among all the U.S. states (21.17% in 2021). The state also expects recurrent hurricanes and extreme heat events frequently^9,38,39^. We answer RQ1 with a quantitative longitudinal study, in which we analyze and compare the impacts of single and compound disasters of hurricanes and extreme heat on older population. The analysis focuses on monthly mortalities caused by heart diseases and strokes in each county of Florida from January 2000 to December 2019 (240 months in total). Along with the variables of climate and weather extremes, we also include (i) the climate variables, (ii) the sociodemographic variables, and (iii) the environment variables in the Poisson regression models to improve the comprehensiveness of the modeling. For RQ2, we develop a set of future *scenarios* that incorporate population growth, climate change, and the occurrence of single or compound disasters in 2050, 2070, and 2100. These prediction periods are adopted by the climate change projections of the U.S. Environment Protection Agency’s (EPA) Locating and Selecting Scenarios Online^40^. With the models calibrated in the longitudinal study (RQ1), we further estimate the monthly mortality caused by heart diseases and strokes among older population in each Florida county under each scenario (RQ2). The predictions can help understand the impacts of climate change and compound disasters on populations’ health at the local and regional levels. The risk assessment results shed light on the planning for the worst-case scenarios under climate change. Such efforts can increase the robustness of adaptation strategies that mitigate the health risks of older adults and other vulnerable populations.

## Results

### Impacts of compound disasters on older population's mortality caused by heart diseases and strokes over the past two decades

**Figure 1:**
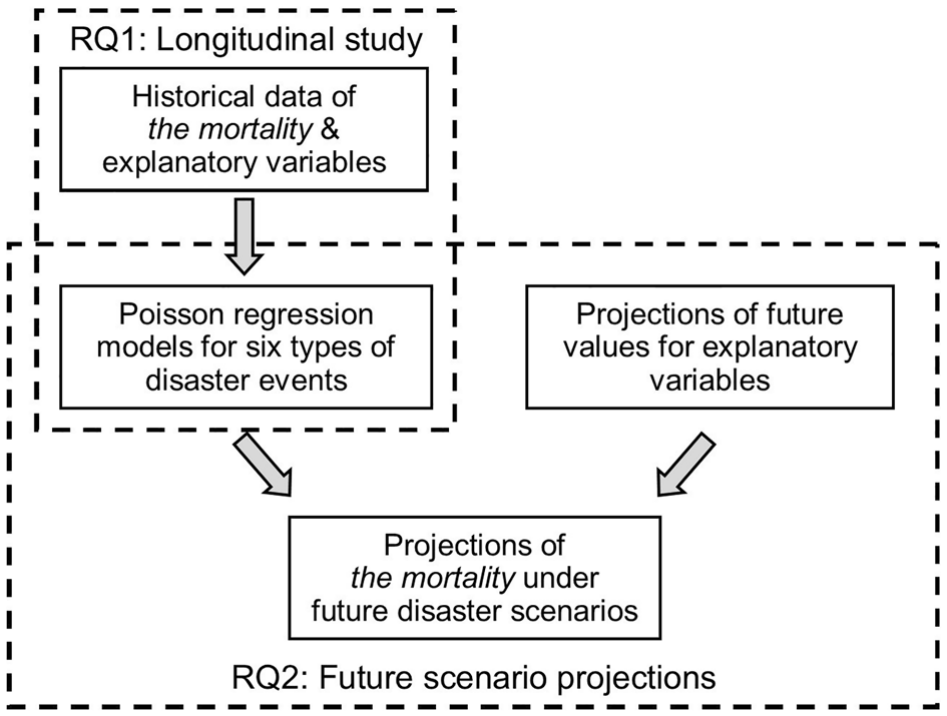
Schematic diagram of the research framework.

**Figure 2:**
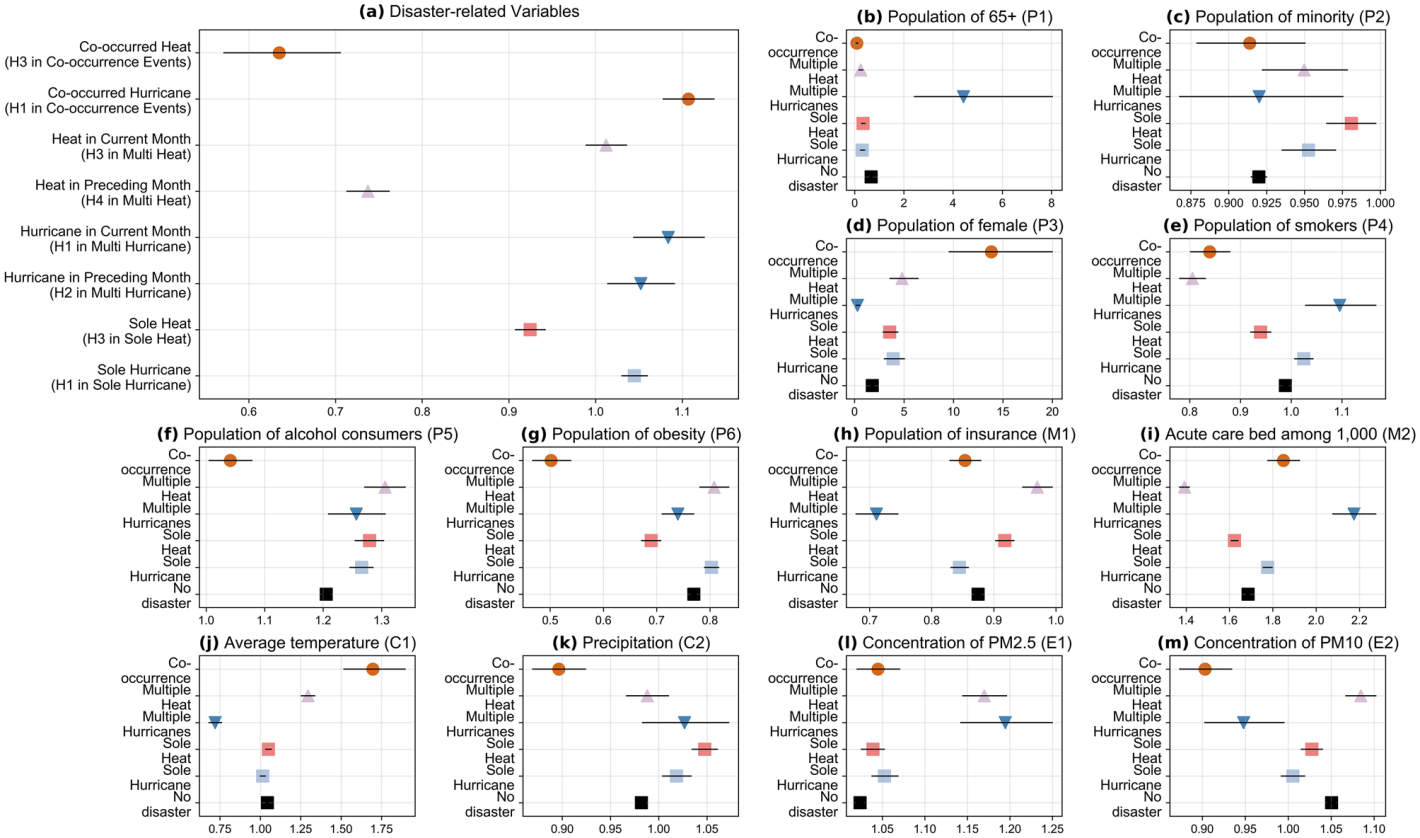
Poisson regression outcomes. The range of coefficient values for disaster-related variables (**a**), the population-related variables (**b–g**), the medical resource availability variables (**h,i**), the climate-related variables (**j,k**), and environmental variables (**l,m**). In each plot, the x-axis shows the estimation and 95% CI for the exponential value of the coefficient. The scatters in each plot indicate the exponential value of the coefficient of the variables under different types of events, and the scope of the dashes indicates the range of 95% CI for the exponential value of the coefficient. The y-axis indicates different types of events, from sole events to co-occurring events (from bottom to top). Especially in (**a**), each type of compound disaster is related to two variables, e.g., “co-occurred hurricane (H1 in co-occurred events)” and “co-occurred heat (H3 in co-occurred events)”. We use the same shape of scatters for variables under the same type of events. Notably, because disaster-related variables have no value in the “No disaster” scenario, we did not include “No disaster” in the y-axis of (**a**).

**Figure 3:**
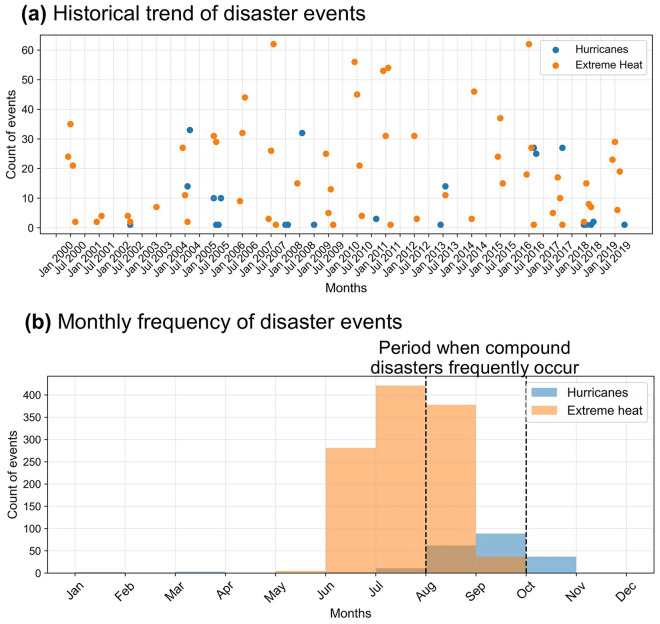
Historical trend (**a**) and monthly frequency (**b**) of hurricane and extreme heat events in Florida from January 2000 to December 2019. Plot (**a**) is a scatter plot, and each scatter represents the count of hurricane or extreme heat events in Florida in a specific month. Plot (**b**) is a histogram, and each bar represents the frequency of hurricane or extreme heat events that happened in a specific month. The compound disasters generally happened between August and October, which is highlighted with dashed lines in the histogram (**b**). Data source: EPA^43^ and NHC^44^.

Following the research procedure shown in Fig. 1, we investigate the impacts of different occurrences of hurricanes and extreme heat (i.e., single and compound disasters) on older populations’ mortality (number of deaths) caused by heart diseases and strokes ( the “*mortality*”) based on Poisson regression analysis (coefficients of regressions under different occurrences of extremes are shown in Fig. 2) with a total of 1326 events of hurricanes and extreme heat and 426 compound disaster events (Fig. 3). Based on the historical records hurricanes and extreme heat events from National Center for Environmental Information and Energy Information Administration^41,42^, hurricanes generally occurred between July and November in the studied period, extreme heat events occurred frequently between May and October. For compound disasters, multi-hurricane events tended to occur between July and October, multi-heat events tended to occur between August and September, and hurricanes and extreme heat tended to co-occur between August and September in the years of the studied period. During the studied period, the overall mortality among older populations in Florida was 917,168, among which the *mortality* of the months with compound disasters was 85,502^18^.

Our Poisson regression models investigate the influence of (i) the occurrence of single or compound disasters, (ii) climate variables, (iii) population variables, and (iv) environmental variables on the *mortality* (variables are listed in Table 1). Especially, we examine the variables’ influence under six event types: (i) no disaster, (ii) single hurricanes, (iii) single extreme heat events, (iv) multiple-hits hurricanes, (v) multiple-hits extreme heat, and (vi) co-occurrence of hurricanes and extreme heat events. The last three types are compound disasters. We regard one variable as more influential if its coefficient is higher in a model calibrated for one type of event than values in other calibrated models.

**Table 1:** Description of factors in the developed scenarios.

Category	Factor	Description	Units	Mean	Max	Min
Climate factors	C1: Monthly average temperature	The monthly average value of recorded temperature within one county	1 °F	70.94	85.4	42.7
	C2: Monthly cumulative precipitation	The monthly cumulative value of recorded precipitation within one county	1 in.	4.42	23.62	0.0
Population factor of older populations (65 or higher ages)	P1: Proportion of older populations	The proportion of the population with ages that are 65 or higher	1%	19.48	87.06	5.38
	P2: Proportion of minority population among older populations	The proportion of older populations who are in minority groups	1%	18.06	58.7	3.8
	P3: Proportion of female older populations	The proportion of the older population population who are female	1%	10.57	50.40	3.07
	P4: Proportion of smokers among older populations	The proportion of the population who are smokers among older populations	1%	9.99	46.2	2.4
	P5: Proportion of alcohol consumers among older populations	The proportion of the population who regularly consume or are addicted to alcoholic drinks among older populations	1%	7.19	41.3	0.1
	P6: Proportion of obesity population among older populations	The proportion of the population whose body-mass index exceeds the threshold of obesity among older populations	1%	25.75	51.8	11.2
Medical resource availability factors	M1: Proportion of older populations without health insurance	The proportion of older populations who have not purchased health insurance	1%	2.54	40.3	0
	M2: Count of acute care beds among 1,000 people	The count of hospital beds for acute care use among every 1000 people within one county	One acute care bed	738.7	7,772	0.0
Environmental factors	E1: Concentration of PM2.5	The annual average value of atmospheric particulate matter (PM) that has a diameter of fewer than 2.5 µm	1 μg/m^3^	1.90	13.9	0
	E2: Concentration of PM10	The annual average value of atmospheric particulate matter (PM) that has a diameter of fewer than 10 µm	1 μg/m^3^	4.14	33.0	0.0
Disaster-related factors	H1: Electricity supply disturbance caused by hurricanes in the current month	Whether the electricity supply system of the local county is disturbed and shut down by hurricanes within one month	1 for “True” and 0 for “False”	Totally 208 events of utility disturbance were caused by hurricanes		
	H2: Electricity supply disturbance caused by hurricanes in the previous month	Whether the electricity supply system of the local county is disturbed and shut down by hurricanes in the previous month	1 for “True” and 0 for “False”			
	H3: Severity of extreme heat events in the current month	The monthly maximum value of recorded temperature within one county	1 °F	Totally 1112 events of extreme heat		
	H4: Severity of extreme heat events in the previous month	The monthly maximum value of recorded temperature within one county in the previous month	1 °F			

In general, the disaster-related variables show significant ($P<0.001$) and positive associations with the *mortality*. By comparing the coefficients (with confident intervals, CI) of disaster-related variables in different calibrated models, we identify that hurricane-induced electricity disturbance and extreme temperatures can influence the *mortality* with different intensities among single-disaster and multi-disaster events. As shown in Fig. 2a, the *mortality* is significantly influenced by the hurricane-induced electricity disturbance (coefficient: 1.045; 95% CI 1.030, 1.060, $P<0.03$) under single-hurricane events. If hurricane-induced electricity disturbances occur in both the current month and the previous month (i.e., forming multi-hurricane events), the *mortality* in the current month is also associated with the disturbance in the previous month. Specifically, under multi-hurricane events, both the electricity disturbance induced by the current-month hurricane can be associated with the *mortality* of the current month with a higher coefficient (coefficient: 1.083; 95% CI 1.044, 1.125, $P<0.001$) than that induced by a single hurricane event (coefficient: 1.045; 95% CI 1.030, 1.060, $P<0.03$). The association between the electricity disturbance induced by the previous-month hurricane and the *mortality* (coefficient: 1.052; 95% CI 1.014, 1.090, $P=0.006$) is also stronger than that induced by a single hurricane event, but such association is weaker than the electricity disturbance induced by the current-month hurricane.

The *mortality* is also significantly associated with the occurrence of single-heat events (coefficient: 0.924; 95% CI 0.908, 0.942, $P<0.001$). If extreme heat also occurs in the previous month and forms multi-heat events, the extreme heat events in the current month can significantly influence the *mortality* in the current month. Specifically, under multiple-heat events, the *mortality* is related to the extreme heat in the current month with a coefficient of 1.012 (95% CI 0.989, 1.036, $P<0.001$), which is slightly higher than the coefficient of extreme heat in sole-heat events (coefficient: 0.924; 95% CI 0.908, 0.942, $P<0.001$). Comparatively, the influence of extreme heat in the previous month on the *mortality* of the current month is much lower than the extreme heat in the single-heat disasters, as the coefficient is only 0.737 (95% CI 0.713, 0.762, $P<0.001$) and the influence is not statistically significant.

When extreme heat and hurricane-induced electricity disturbance co-occur in the same month, the electricity disturbance can still influence the *mortality* in the current month significantly (coefficient value: 1.107, 95% CI 1.078, 1.137, $P<0.001$), which is slightly higher than their influence under sole-hurricane events (coefficient: 1.045; 95% CI 1.030, 1.060, $P<0.03$). Differently, the influence of extreme heat on the *mortality* is much lower than the impact of extreme heat under single-heat events. Specifically, when co-occurring with hurricane-induced electricity disturbance, the extreme heat can influence the *mortality* in the current month with a coefficient of 0.635 (95% CI 0.571, 0.705, $P<0.001$), which is much lower than the coefficient under single-heat events (coefficient: 0.924; 95% CI 0.908, 0.942, $P<0.001$).

Meanwhile, the associations between the *mortality* and other variables also demonstrate some notable trends. The proportion of the older population (Fig. 2b), the proportion of smokers among older populations (Fig. 2e), and the availability of acute care beds (Fig. 2i) tend to have notable correlations with the *mortality* under weather extremes when multiple-hurricane events occur. The proportion of the minority population among older populations (Fig. 2c), the proportion of alcohol consumers among older populations (Fig. 2f), the proportion of obese people among older populations (Fig. 2g), the proportion of people without health insurance among older populations (Fig. 2h), and the concentration of PM10 (Fig. 2m) tend to have notable correlations with the *mortality* when multi-heat events occur. The proportion of females (Fig. 2e) and the average air temperature (Fig. 2k) tend to associate with the *mortality* with higher impacts during compound disaster events.

### Anticipating older populations’ health risks under the developed scenarios based on the mortality caused by heart diseases and strokes

**Figure 4:**
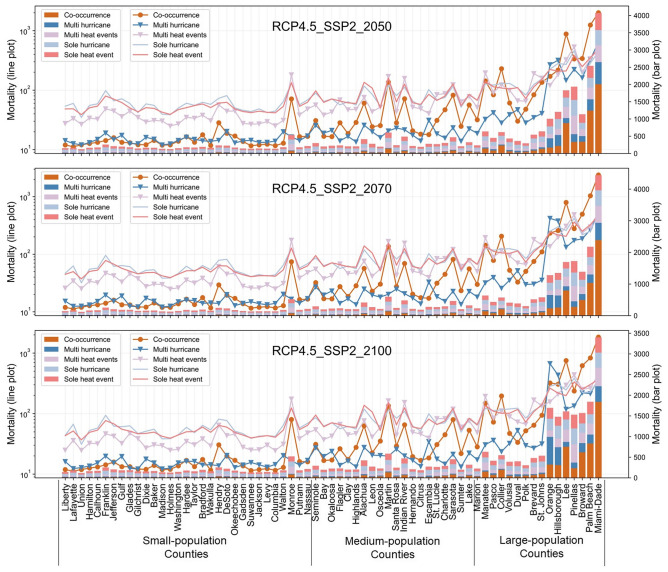
Projected monthly mortality caused by heart diseases and strokes in each county of Florida under future scenarios (RCP4.5, SSP2). The x-axis indicates the counties of Florida, which are divided into three groups to facilitate the comparison of predicted mortality levels with counties with similar levels of populations. Counties from left to right are ordered based on the population size. Specifically, counties with less than 100,000 people are classified as “small-population counties”, counties with 100,000 to 500,000 people are “medium-population counties”, and counties with more than 500,000 people are “large-population counties”. The y-axis on the left is scaled for the value of projected mortality (represented by scatters and lines), helping to illustrate the difference between small-population counties and large-population counties. The y-axis on the right indicates the value of projected mortality without being scaled (represented by bars).

**Figure 5:**
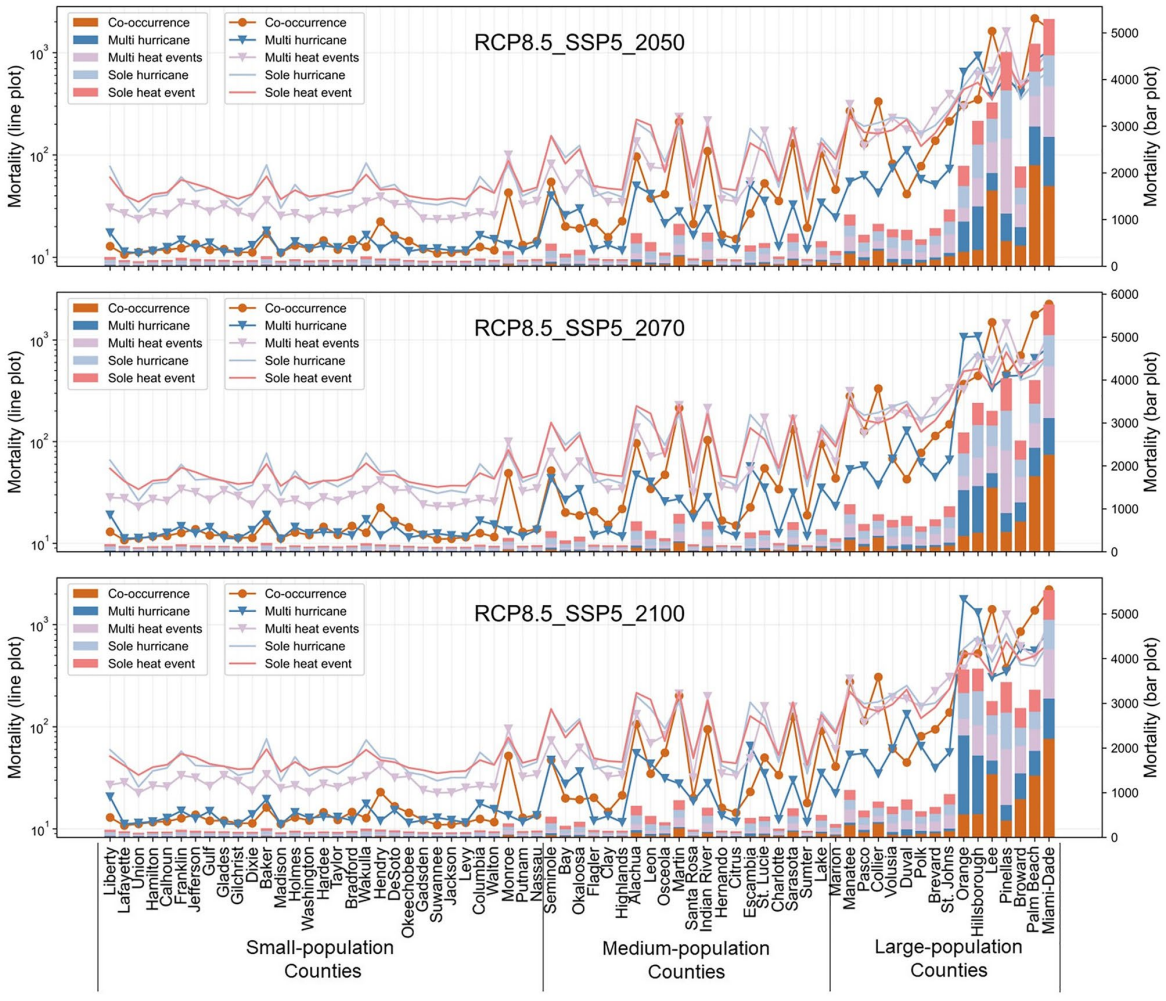
Projected monthly mortality caused by heart diseases and strokes in each county of Florida under future scenarios (RCP8.5, SSP5). The x-axis indicates the counties of Florida, which are divided into three groups to facilitate the comparison of predicted mortality levels with counties with similar levels of populations. Counties from left to right are ordered based on the population size. Specifically, counties with less than 100,000 people are classified as “small-population counties”, counties with 100,000 to 500,000 people are “medium-population counties”, and counties with more than 500,000 people are “large-population counties”. The y-axis on the left is scaled for the value of projected mortality (represented by scatters and lines), helping to illustrate the difference between small-population counties and large-population counties. The y-axis on the right indicates the value of projected mortality without being scaled (represented by bars).

Based on the relationships between the occurrence of single and compound disasters and the *mortality*, we anticipate older populations’ mortality caused by heart diseases and strokes (RQ2) under future scenarios in 2050, 2070, and 2100 (Figs. 4, 5). The future scenarios are developed considering (i) the occurrence of single and compound disasters, (ii) the changing climate, and (iii) the trend of population growth (illustrated in Fig. 7). The x-axis represents the 67 counties of Florida, and the order of the counties is based on the size of their older populations in 2019. The counties on the right side of the figures have a larger population of older populations than the counties on the left. The absolute value of the estimated monthly mortality of older populations due to heart diseases and strokes in each county is shown with the bars. We also draw lines based on the log-base-ten value of the anticipated mortality to facilitate a comparison of the *mortality* between large-population and small-population counties. To facilitate robust planning that considers both the mid-of-way and worst-case scenarios, we compare the *mortality* of different scenarios under RCP4.5-SSP2 and RCP8.5-SSP5. Specifically, “RCPs” stand for “representative concentration pathways”, indicating the greenhouse gas concentration levels that are measured by the radiative forcing levels in 2100^27^. “RCP4.5” represents that the greenhouse gas concentration will lead to the radiative forcing of 4.5 Watts per meter squared in 2100, indicating the middle-of-way emission level^27^. “RCP8.5” that the greenhouse gas concentration will lead to the radiative forcing of 8.5 Watts per meter squared in 2100, indicating the high emission level^28^. “RCPs” are the climate change trajectories and the basis of existing climate models when projecting the future range of climate variables (e.g., temperature and precipitation). Meanwhile, “SSPs” stand for “shared socioeconomic pathways”, i.e., trajectories of socioeconomic developments that are characterized by population growth and economic development under future climate change scenarios^43^. There are five SSPs, i.e., SSP1 to SSP5^43^. Among the five SSPs, SSP2 represents middle-of-the-road development (e.g., middle-level population growth), while SSP5 represents fossil-fueled development (e.g., high-level population growth)^43^. The scenarios of RCP4.5-SSP2 can show the potential mortality under different weather extreme events, with sustainable pathways for population growth and greenhouse gas emissions. On the contrary, the scenarios of RCP8.5-SSP5 show the potential mortality under different weather extreme events, with rapid population growth and the high greenhouse gas emissions associated with aggressive fossil-fuel use.

Under the scenarios with the same combination of RCPs and SSPs (i.e., within the same figure of Figs. 4, 5), our anticipated outcomes show that the co-occurrence of hurricanes and extreme heat is expected to be concurrent with markedly higher-level mortality of older populations from heart diseases and strokes compared to the other weather extreme events in most counties, especially the counties with a large population of older populations. For example, in Miami-Dade County (459,200 older adults in 2019), the co-occurrence of hurricanes and extreme heat under RCP4.5-SSP2 can be accompanied by older populations’ mortality caused by heart diseases and strokes that will reach 1800 for one month in 2100, which is significantly higher than the *mortality* under scenarios with other weather extreme events. Despite the co-occurrence of hurricanes and extreme heat, multi-hurricane and multi-heat events are expected to cause the *mortality* at the same level as the co-occurred weather extreme events, especially in medium-population counties. For example, in Indian River County (51,300 older adults in 2019), the multiple-heat events and the co-occurrence of heat and hurricanes are expected to bring the *mortality* to similar levels under RCP4.5-SSP2 in 2050, 2070, and 2100. Also, in Seminole County (73,300 older adults in 2019), multiple heat events are expected to cause monthly mortality of 150 in 2100 under RCP8.5-SSP5, which is much higher than the expected monthly mortality under the impact of the co-occurrence of extreme heat and hurricanes in 2100 under RCP8.5-SSP5 (45 mortality).

We also compare the effects of the same weather extreme events under different combinations of RCPs and SSPs (i.e., comparing across Figs. 4, 5). Our comparisons indicate that most events of weather extremes tend to be simultaneous with more severe mortality caused by heart diseases and strokes among older populations under RCP8.5-SSP5 compared to RCP4.5-SSP2 (with averagely 20.5% of the increase), especially under multi-hurricane events and co-occurred hurricanes and heat events. Taking Miami-Dade County as an example, the *mortality* is expected to reach 365 under multi-hurricane events, RCP4.5-SSP2 in 2100. Comparatively, multi-hurricane events can potentially increase the monthly mortality to 893 (144.65% increase) in 2100 under RCP8.5-SSP5. Also, under RCP4.5-SSP2 in 2100, the *mortality* is expected to be around 1800 in Miami-Dade County if hurricanes and extreme heat events co-occur, while the *mortality* may increase to around 2200 under RCP8.5-SSP5 in 2100 (22.2% increase). Overall, with the tremendous increase of population and temperature rising in RCP8.5-SSP5, compound disasters (i.e., multi-disaster events or co-occurring disasters) can potentially intensify older populations’ mortality caused by heart diseases and strokes to a level that is much higher than the expected mortality under middle-of-way scenarios.

## Discussion

Our longitudinal study and future-scenario anticipations indicate that older populations’ risks of heart diseases and strokes can potentially be intensified under compound disasters of hurricanes and extreme heat events. We find that under multi-hurricane events in two continuous months, the older populations’ mortality (number of deaths) within the same county caused by heart diseases and strokes can significantly increase when hurricanes occur in the second month. The influence of both two hurricane events is also more intense than the impact of sole-hurricane events. When extreme heat occurs in two consecutive months, the extreme heat of the second month can influence the monthly mortality of the second month with a higher effect than that of sole-heat events. Additionally, in the same county, under future scenarios with the same combination of RCPs and SSPs, the *mortality* is expected to be higher under the co-occurrence of hurricanes and extreme heat than the occurrence of other types of weather and climate extremes. This condition is particularly pronounced in counties hosting substantial aging populations. Most weather extreme events tend to be accompanied by a larger number of deaths caused by heart diseases and strokes among older populations under RCP8.5-SSP5 compared to RCP4.5-SSP2, especially with multi-heat events and the co-occurrence of hurricanes and heat. Our findings can provide compelling evidence regarding the association between compound disasters and the older population’s mortality and inform future strategies for risk mitigation.

Our study goes beyond previous studies in revealing the health impacts of compound disasters from multiple aspects. First, existing studies have mainly focused on the health impacts of weather extremes on the general population, lacking sufficient attention to older adults, who are among the most vulnerable to the negative effects of a rapidly changing climate^4,7,44^. To fill this gap, we focus on older populations and estimate the potentially high-level impacts of future weather extremes on their health. Our findings highlight the necessity of investigating the health risks of different groups of older populations to promote a comprehensive understanding of the impacts of climate change and weather extremes.

Second, the results of existing studies on compound disasters mainly involve limited cases, e.g., Hurricanes Rita and Katrina^33^ and the co-occurrence of the COVID-19 pandemic and Hurricane Ida^25^. The findings from these limited cases may not provide a reference for understanding the impacts of other types of weather extremes, such as multi-heat events. Comparatively, the temporal and spatial scales of our longitudinal study are much broader, covering hurricanes and extreme heat events in 67 Florida counties over 20 years. With sufficient numbers of both single-disaster events and compound disasters, our longitudinal study can contribute to promoting the understanding of the associations between older populations’ health risks and different weather extremes under more uncertain and diverse circumstances. Communities with similar context, e.g., other counties in the Southeastern U.S. confronting extreme heat and tropical cyclones, may use our findings as a reference for understanding the impacts of climate change locally. Our longitudinal findings of compound disaster events can also provide some insights regarding how the influence of multiple hazards can be altered when they are compounded in a narrow temporal and spatial frame.

Third, the existing literature has rarely anticipated the health consequences of compound disasters under future scenarios^45^. Findings solely based on historical cases without considering the uncertain trends of climate change and population growth^12^, such as Cherry et al.^33^ and Fuhrmann et al.^3^, may not reflect the impact of compound disasters under future scenarios. Our scenario-based anticipation of older populations’ mortality caused by heart diseases and strokes fills this gap and provides a more intuitive estimation of the plausible risk levels of specific counties under single or compound disasters. The anticipated outcomes can provide a basis for developing regional and community strategies for both the “business-as-usual” situations and the “worst-case” compound disasters. The cross-scenario assessment framework of vulnerable populations’ health risks can guide local communities and researchers to specify the future scenarios they may confront and facilitate them to disentangle the uncertain impacts of future climate change and weather extremes on local populations and the built environment.

Our study has some limitations that present opportunities for future research. First, our longitudinal findings may not apply to scenarios where temperature and precipitation are outside the range covered by our historical data and the employed climate projections. For example, the monthly maximum temperature in our historical data and employed climate projections were below 98 Fahrenheit degrees, but climate change may result in more extreme temperatures^44^. The relationship between the temperature of such extreme values and the *mortality* may deviate from our longitudinal findings. Future studies could employ datasets with broader value ranges of temperature and precipitation to capture more accurate relationships between these factors and the *mortality*. Second, we measured the health risks and sociodemographic characteristics of older populations at the population level, while the individual-level characteristics of older adults were not studied. Future studies can leverage individual-level information and characteristics, such as medical insurance records and social media data^46,47^, to reveal the compounding influence of social determinants of health on older adults’ health risks under climate change. However, our projections of the mortality at the population level can still provide a macro assessment of the health impacts caused by large-scale hazard events. Third, we developed future scenarios based on current projections for climate change and population growth from EPA^40^ and Hauer^48^, while the projected population may not be reached under the increase of projected temperature. Older populations’ relocation behaviors motivated by the increasing temperature and their changing capacity to adapt to high-temperature weather may result in different population pathway^49,50^. Future studies may adopt more up-to-date projections of population growth and climate change for estimating the *mortality* of older population. Fourth, additional environmental factors, such as air pressure and sea surface temperature, may also influence older adults’ health risks under future climate change^28^, but were not included in our assessments. Specifically, we considered the climate variables (i.e., temperature and precipitation) as suggested by existing climate projection models, such as the Geophysical Fluid Dynamic Laboratory (GFDL) and the Hadley Centre Global Environment Model (HadGEM2)^51,52^. Also, the regional-level air quality change we adopted in the future scenarios may not accurately reflect air quality at sub-regional scales, such as the county level^53,54^. Future studies could utilize local data collection and place-based projections to address the needs of subregional risk assessment. Additionally, this study focuses on compound disasters of hurricanes and/or extreme heat in Florida. Future studies can extend the investigation to other combinations of disaster types in other geographic regions, such as wildfire events and droughts in California.

Hurricanes and extreme heat events are expected to exacerbate older populations’ mortality caused by heart diseases and strokes severely if they occur as compound disasters. Our study reveals the potentially exacerbated risks of heart diseases and strokes among the older populations under the future scenarios of climate change and compound disasters. The results highlight the interactions between different weather extremes that form compound disasters, which can amplify the influence of specific weather extremes (e.g., hurricanes) on the older populations’ health risks significantly. Our findings provide a reference for developing robust risk mitigation strategies that are targeted to the health threats of different occurrences of weather extremes. Specifically, based on our scenario-based anticipations, local communities can develop risk mitigation strategies that are tailored for both single and compound disaster scenarios, avoiding the potential overlapping of disaster preparedness, response, and recovery. Our findings also suggest strengthening the collaboration among the facilities of public health, lifeline services, and disaster management, designing cross-departmental risk mitigation strategies for maintaining vulnerable populations’ access to medical services during compound disasters. By comprehensively capturing the uncertain threats of compound disasters to the human population, adaptation strategies can mitigate the health risks and improve the well-being of older populations and other vulnerable populations effectively.

## Methods

### A longitudinal study of compound disasters’ impacts on older populations’ mortality caused by heart diseases and strokes

Florida has the second-highest percentage of senior citizens among U.S. states (21.17% in 2021) and expects recurrent hurricanes and extreme heat events frequently^9,38,39^. Older populations’ mortality (number of deaths) caused by heart diseases and strokes in Florida is high, reaching 50,616 deaths in 2019^18^. The longitudinal study was conducted with the monthly data of 67 Florida counties from January 2000 to December 2019, as 2000 was the first year when the monthly data of the mortality caused by heart diseases and strokes in each county of Florida became available. The data since 2020 when the COVID-19 pandemic started was not considered, as the pandemic can disturb the trend of older populations’ mortality in counties of Florida. The longitudinal data on older populations’ monthly mortalities caused by heart diseases and strokes in Florida was obtained from the open-access datasets collected by the CDC WONDER. Our study did not contain any experiments with human participants or animals and followed the data usage policies of FDOH. An “event” represents the occurrence of weather extremes in one county within one month. Each event includes the records of the *mortality*, climate characteristics (temperature and precipitation), air quality, population characteristics, and the type and intensity of weather extreme events. As the events have a short time step, i.e., one month, the longitudinal study based on the events can capture the immediate impacts of extreme heat events and hurricanes on older populations’ mortality caused by heart diseases and stroke^55,56^.

The longitudinal study covered six types of weather extreme occurrences and compared their impacts on older populations’ mortality caused by heart diseases and strokes, including (i) no disaster, (ii) single hurricanes, (iii) single extreme heat events, (iv) multiple-hits hurricanes, (v) multiple-hits extreme heat, and (vi) co-occurrence of hurricanes and extreme heat events. The last three types are compound disasters. The historical records of hurricanes and extreme heat events were collected from the U.S. National Center for Environmental Information (including the records of extreme heat) and the U.S. Energy Information Administration (including the records of hurricane-induced power outages)^41,42^. “Multiple hits” disasters are defined as several weather extremes of the same type affecting the same county in two continuous months. The “co-occurrence” refers to the events in which hurricanes and extreme heat affect the same county within the same month. The events were extracted from the monthly data of 67 Florida counties and categorized into six groups based on the six types of weather extreme occurrences. For each group of events, we quantified the impacts of each type of weather extreme event on older populations’ mortality caused by heart diseases and strokes respectively. Especially, the severity of hurricanes and extreme heat events was represented by the occurrence of hurricane-induced electricity disturbance and monthly maximum temperature. The occurrence of hurricane-induced electricity supply disturbance was set with a binary value, i.e., 0 for “not occurring” and 1 for “occurring”. To represent the severity of extreme heat, we used 0 to represent the situation in which no extreme heat events occur, and we represented the severity with the absolute value of the monthly maximum temperature if extreme heat events occur.

The longitudinal study is based on multivariate Poisson regression (Eq. 1). ${\beta }_{0}$ refers to the constant, and ${\beta }_{m, n}$ (e.g., ${\beta }_{1, i}$) refers to the coefficient of each independent variable in the Poisson regression. Poisson regression can explain the statistical relationships between multiple factors and the count of small-probability events occurring (e.g., disease-induced mortality)^57^. Poisson regression has been utilized to capture the association between the *mortality* of heat-related illnesses and specific determinants^45,58^. The dependent variable is the county-level monthly mortality c heart diseases and strokes among older populations ($HD$), i.e., the older adults who died because of heart diseases or strokes in a specific month within a specific county. The independent variables in our Poisson regression models are described in Table 1. Each of these variables shows a relatively independent nature from the others while exhibiting discernible correlations with the dependent variable, as depicted in Supplementary Figs. S1 and S2. Specifically, the impact of weather extreme events is represented by the disaster-related variables (i.e., ${H}_{n}$), covering the occurrence of hurricane-induced electricity supply disturbance and the monthly maximum temperature. Both variables can represent the intensity of hurricanes and extreme heat on influencing the risks of heart disease and stroke among older populations^59,60^. We did not include variables representing the duration of weather extreme events, which may not reflect the duration that older adults were exposed to the weather extremes in the studied period^27^. The explanatory variables also include the climate variables (${C}_{i}$, including the average temperature and cumulative precipitation), air pollution (${E}_{m}$, including the concentrations of PM2.5 and PM10), population factors (${P}_{j}$), and health resource availability (${M}_{k}$) as the explanatory variables. Specifically, the population factors include the proportion of older populations, the proportion of minority populations among older populations, the proportion of females among older populations, the proportion of smokers among older populations, the proportion of alcohol consumers among older populations, and the proportion of obesity population among older populations^61,62^. We selected these variables because according to the “Heart Disease and Stroke Statistics—2022 Update” from the American Heart Association, the risks of heart disease and stroke were significantly different among populations of different genders, minority groups, and obesity^63^. Also, the use of alcohol was the leading behavioral factor of mortality caused by heart disease and stroke^63^. We also considered the proportion of people with health insurance among older populations and the count of acute care beds among 1,000 people to represent the accessibility of health resources among older populations. For each type of weather extreme occurrence, we trained specific Poisson regression models based on the historical records of that weather extreme occurrence and the value of independent variables in related months (the historical trend of each independent variable is shown in Supplementary Fig. S3).

1 $$\mathrm{log}\left(HD\right)={\beta }_{0}+\mathrm{exp}(\sum \limits_{i=1}^{2}{\beta }_{1,i}{C}_{i}+\sum \limits_{j=1}^{6}{\beta }_{2,j}{P}_{j}+\sum \limits_{k=1}^{2}{\beta }_{2,k}{M}_{k}+\sum \limits_{m=1}^{2}{\beta }_{4,m}{E}_{m}+\sum \limits_{n=1}^{4}{\beta }_{5,n}{H}_{n}).$$

### Developing disaster scenarios for anticipating future older population's mortality caused by heart diseases and stroke

**Figure 6:**
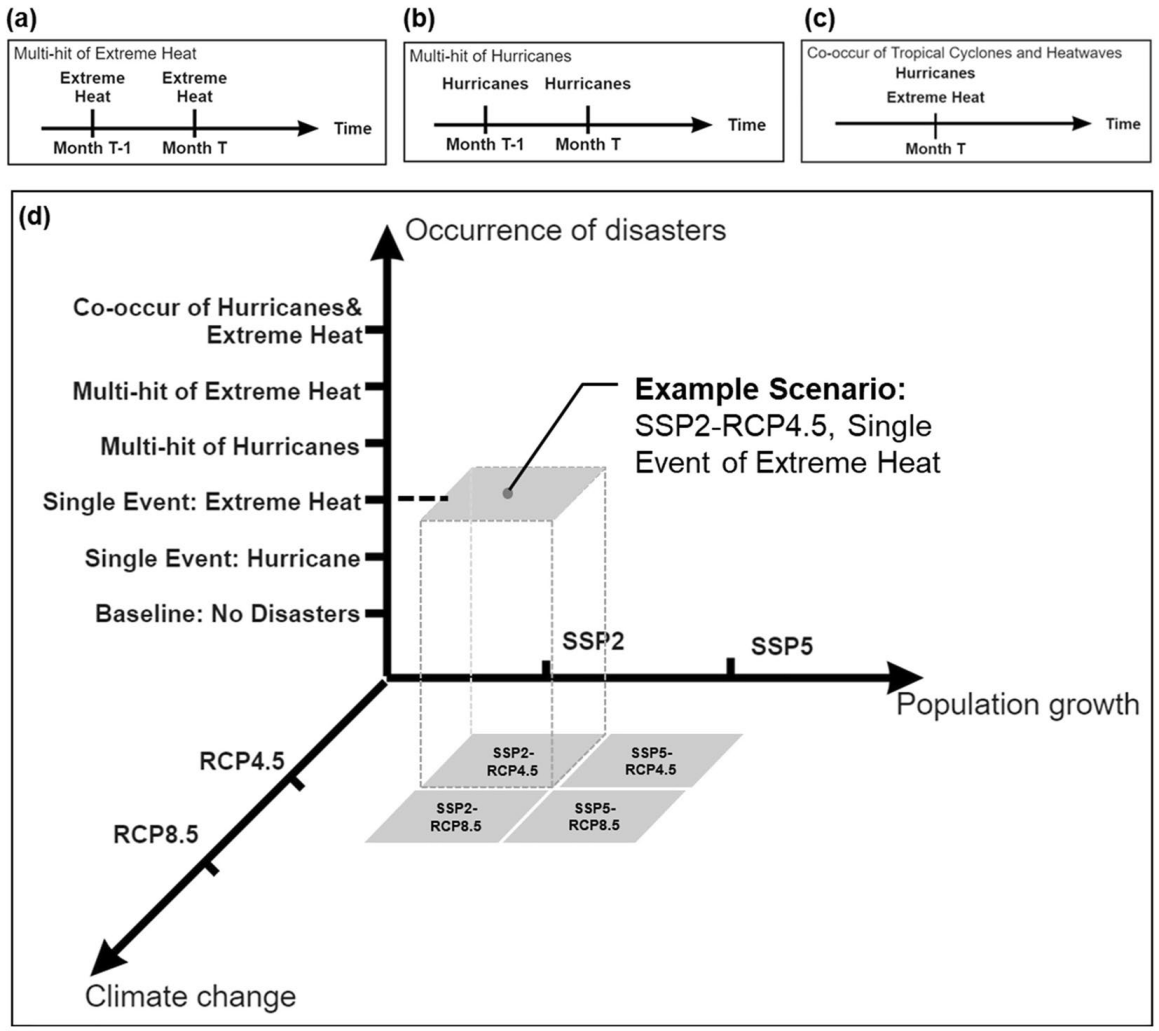
Three types of compound disasters (**a–c**) and scenario development across climate change, population growth, and the occurrence of weather extremes. Plots (**a,b,d**) are illustrations of the three types of compound disasters: multi-hit of extreme heat, multi-hit hurricanes, and co-occurring hurricanes and extreme heat. Plot (**d**) shows the three dimensions of future scenarios. The grey squares in the bottom surface indicate the four types of climate change pathways in the United States, including “SSP2-RCP4.5”, “SSP5-RCP4.5”, “SSP2-RCP8.5”, and “SSP5-RCP8.5”. Plot (**d**) also includes an example scenario, which is linked to the climate change pathways and occurrences of weather extremes with dash lines.

The calibrated models of longitudinal studies are the basis for anticipating older populations’ mortality caused by heart diseases and strokes at the county level under future scenarios over 2050, 2070, and 2100. We adopted the prediction periods of the U.S. Environment Protection Agency’s (EPA) Locating and Selecting Scenarios Online^40^, i.e., 2050, 2070, and 2100. The future scenarios incorporate the dimensions of climate change, population growth, and the occurrence of weather extremes (Fig. 6). We considered different combinations of the three dimensions in the developed scenarios, aiming to capture the possible range of older populations’ mortality caused by heart diseases and strokes in the uncertain future.

**Figure 7:**
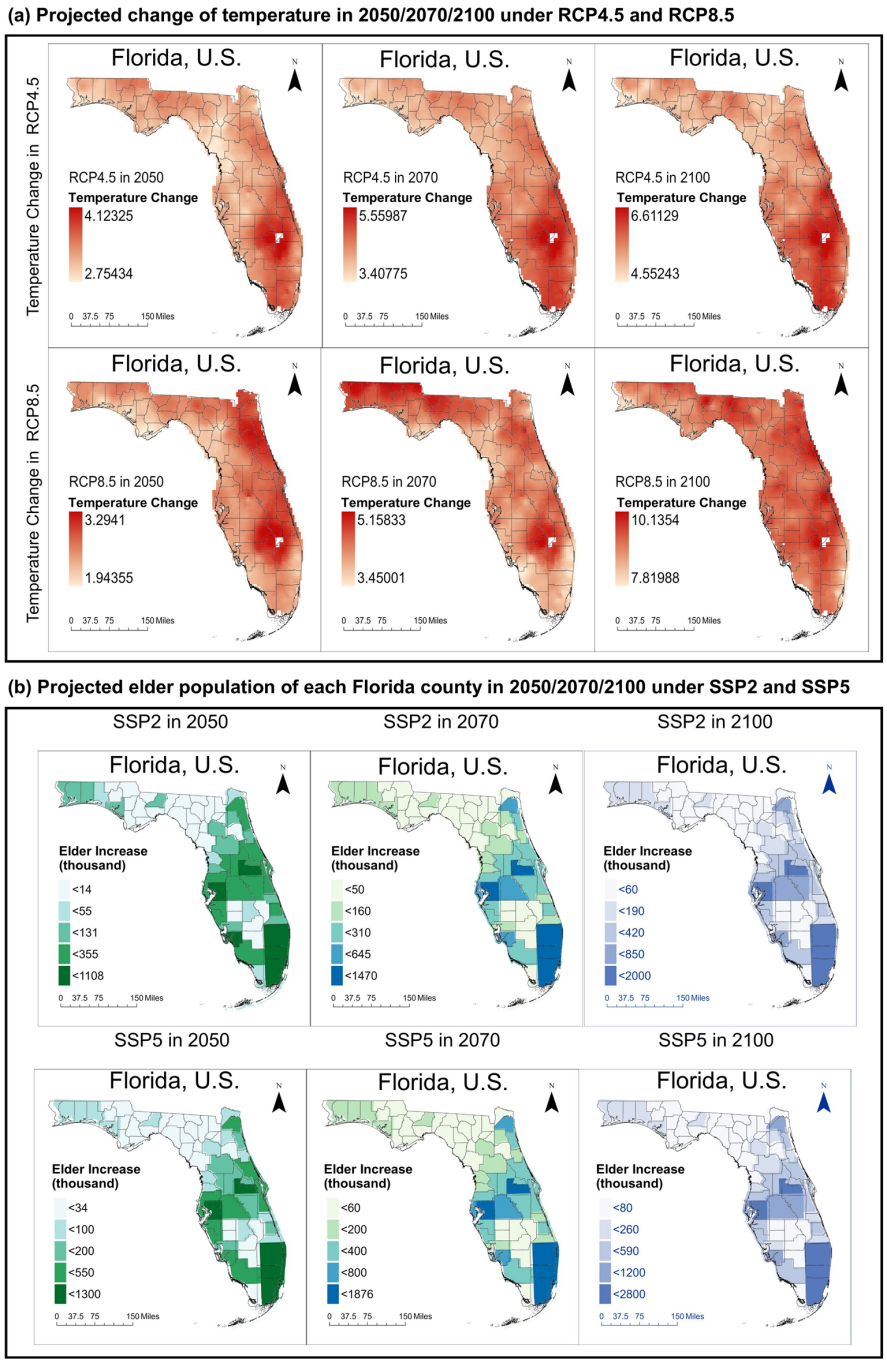
Projection data of (**a**) temperature and (**b**) older population growth. The darker colors represent the higher values of temperature change and population growth. This figure is produced using ArcGIS Pro Version 2.5, provided by ESRI (https://www.esri.com/en-us/arcgis/products/arcgis-pro/trial). Data source: EPA^40^ and Hauer^41^.

Similar to the longitudinal study, future scenarios cover six event types (the values of disaster-related variables are shown in Table 2). The severity of future climate and weather extremes is represented by the occurrence of hurricane-induced electricity supply disturbance and maximum temperature. The climate change in our scenarios is represented by the change in local average temperature, precipitation, and air pollution concentrations under RCPs^40,53,54^. Specifically, the temperature and precipitation change are estimated based on the projections from the Geophysical Fluid Dynamic Laboratory (GFDL) under CMIP5-based RCP4.5 and RCP8.5 (shown in Fig. 7a)^47^. The resolution of the projections is 1/16°, i.e., around 6.9-km resolution^40^. We calculated the average values of temperature change and precipitation change among the land cells within each county. For the air pollutant concentration, there is no widely accepted projection about the fine-scale air quality change under RCPs in Florida from 2050 to 2100. Based on the projected trends of air pollution concentrations under climate change^53,54^, we set the volume of air pollutant concentration under RCP4.5 as zero (i.e., air pollutants can be ignored) to represent the low-emission scenarios under the future emission controls. Under RCP8.5, we set the volume of air pollutant concentration as the same as in 2019, representing the business-as-usual scenarios. For the population growth, we adopted Hauer's projections^48^ of the county-level population of older adults (Fig. 7b), minority, and female older populations. We can identify an increase trend of older populations under both the scenarios of SSP2 and SSP5 in 2050, 2070, and 2100. We adopted the basic assumptions of population growth under SSP2 and SSP5 in Hauer’s projections^48^. Specifically, for high-income OECD countries (e.g., the U.S.), the population growth under SSP2 could follow the medium levels of fertility, mortality, and migration in historical records. In contrast, the population growth under SSP5 could follow the high levels of fertility and migration, while the mortality levels would be low levels based on historical records. Particularly, the increasing trend of older populations under SSP5 would be more tremendous than the increasing trend under SSP2. For example, the older population in Miami-Dade County is projected to increase by 1.996 million under SSP2 in 2100, while the projection is 2.781 million under SSP5 in 2100. The increasing trend of the older population highlights the necessity of investigating their health risks under future scenarios. Additionally, we regarded the proportion of smokers, alcohol consumers, population with health insurance, the obese population, and the count of acute care beds among 1000 population in the future scenarios as the same as the value in 2019. All the assumptions we adopted when applying regression outcomes to future scenarios are listed in Supplementary Table S1.

**Table 2:** Values of disaster-related factors.

Occurrence of weather extreme s	${H}_{1}$	${H}_{2}$	${H}_{3}$	${H}_{4}$
No disaster	0	0	0	0
Sole hurricane	1	0	0	0
Sole heat events	0	0	Current max temperature	0
Multi hurricane	1	1	0	0
Multi heat events	0	0	Current max temperature	Past max temperature
Co-occurrence	1	0	Current max temperature	0

**Figure 8:**
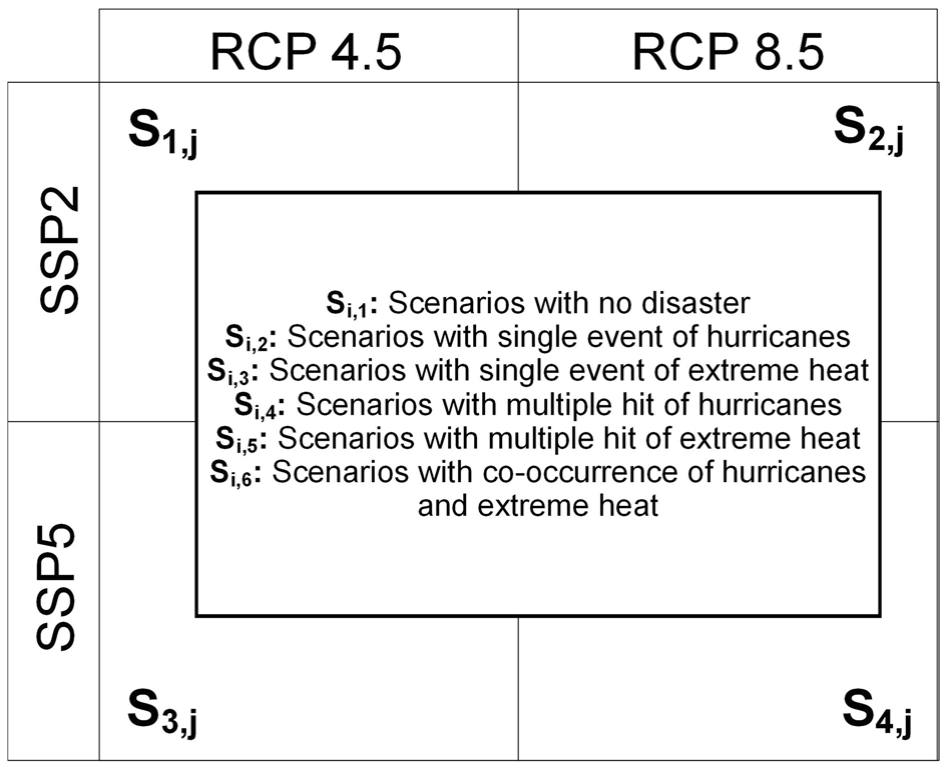
Developed scenarios: ${S}_{i,j}$. $i$ represents the combinations of RCPs and SSPs, $j$ represents the types of weather extreme occurrences.

With the three dimensions in Fig. 6, we developed a set of future scenarios (shown in Fig. 8). Each scenario is assigned a unique code ${S}_{i,j}$. $"i"$ of ${S}_{i,j}$ represents the combination of SSPs and RCPs (e.g., ${S}_{1,j}$ represents scenarios under RCP4.5-SSP2), and $"j"$ of ${S}_{i,j}$ represents the type of weather extreme occurrences (e.g., ${S}_{i,6}$ represents scenarios of co-occurred hurricanes and extreme heat). Overall, the scenarios are under the four combinations of SSPs and RCPs: SSP2-RCP4.5, SSP2-RCP8.5, SSP5-RCP4.5, and SSP5-RCP8.5. We assigned one of the six event types for each scenario, represented by $"j"$ of ${S}_{i,j}$. Using the calibrated Poisson regression models in the previous section, we estimated the monthly mortality caused by heart diseases and strokes among older populations in each Florida county under each of the developed scenarios. We compared the anticipated mortality levels across scenarios to investigate if higher-level mortality caused by heart diseases and strokes among older adults occurs in compound disasters than in single extreme events.

### Supplementary Information

Supplementary Information.

## Data Availability

For the datasets of historical records, we collected the older populations’ monthly mortality related to heart diseases and strokes for each county in Florida, U.S. from the public-accessed dataset of the CDC WONDER (https://wonder.cdc.gov/). The historical records of Florida counties’ older populations were collected from the Florida Department of Health (https://www.flhealthcharts.gov/FLQUERY_New/Population/Count). The historical records of the local temperature (including extreme heat) and air quality in Florida counties were collected from National Centers for Environmental Information (https://www.ncei.noaa.gov/access/monitoring/climate-at-a-glance/county/time-series) and U.S. Environmental Protection Agency (https://www.epa.gov/outdoor-air-quality-data). The occurrence of the hurricane-induced power outage was collected from the U.S. Energy Information Administration (https://www.eia.gov/electricity/monthly/). The historical data on population-related factors in each county of Florida were collected from the Florida Department of Health (https://www.flhealthcharts.gov/FLQUERY_New/Population/Count# and https://www.floridahealth.gov/statistics-and-data/survey-data/behavioral-risk-factor-surveillance-system/index.html). The historical data of “acute care beds per 1000 people” was collected from the Florida Department of Health (https://www.flhealthcharts.gov/ChartsDashboards/rdPage.aspx?rdReport=NonVitalIndNoGrp.Dataviewer&cid=0314). For the datasets utilized for future scenarios, the future population levels of Florida counties are based on the population projections developed by Mathew E. Hauer (https://www.nature.com/articles/sdata20195). The future temperature change is based on the U.S. Environmental Protection Agency’s predictions (https://lasso.epa.gov/). We inferred future air quality and occurrence of extreme events in our developed scenarios as described in the “Methods” section. The datasets generated in the current study are available from the corresponding author upon reasonable request.
