# Supplementary material for: Anticipating older populations’ health risk exacerbated by compound disasters based on mortality caused by heart diseases and strokes
Source: Sci Rep. 2023 Oct 5;13:16810. doi: 10.1038/s41598-023-43717-3 (PMC10556062; doi:10.1038/s41598-023-43717-3)
Supplement: Supplementary file 1 — Supplementary Information. [file 41598_2023_43717_MOESM1_ESM.docx]

**Anticipating Older Populations’ Health Risk Exacerbated by Compound Disasters Based on Mortality Caused by Heart Diseases and Strokes**

**Shangde Gao^1^, Yan Wang, Ph.D.^1^***

^1^Department of Urban and Regional Planning and Florida Institute for Built Environment Resilience, University of Florida, Gainesville, FL, 32611, USA.

*yanw@ufl.edu

**Supplementary Figure S1** Pair correlation between the explanatory variables


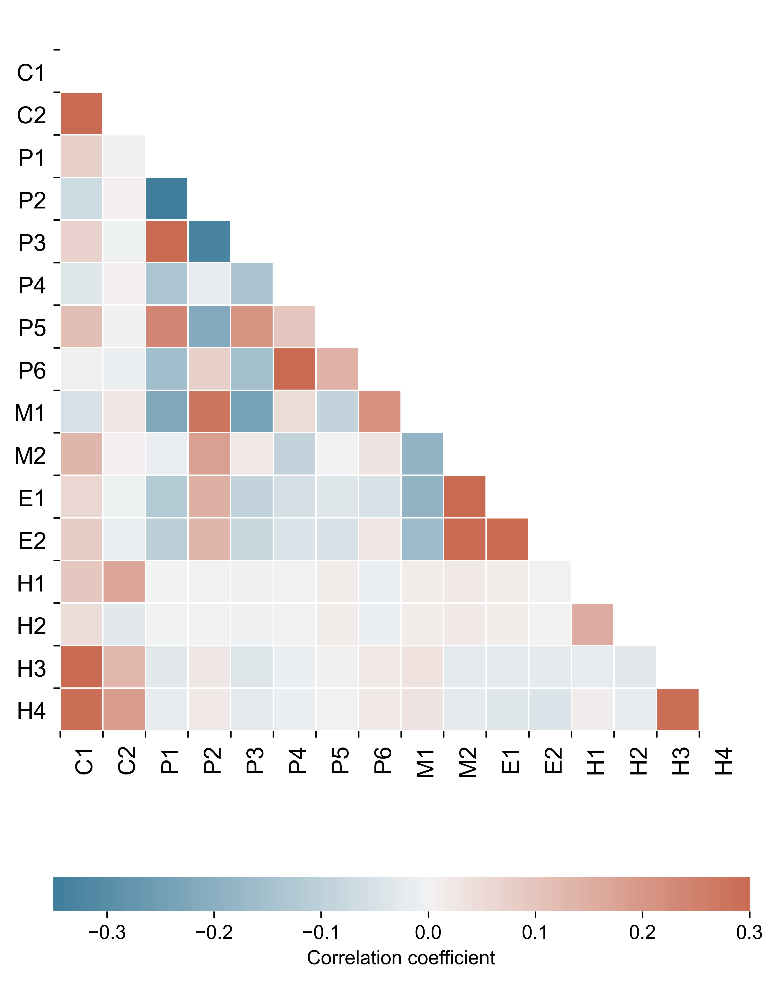


**Supplementary Figure S2** Correlations between each explanatory variable and the *mortality*


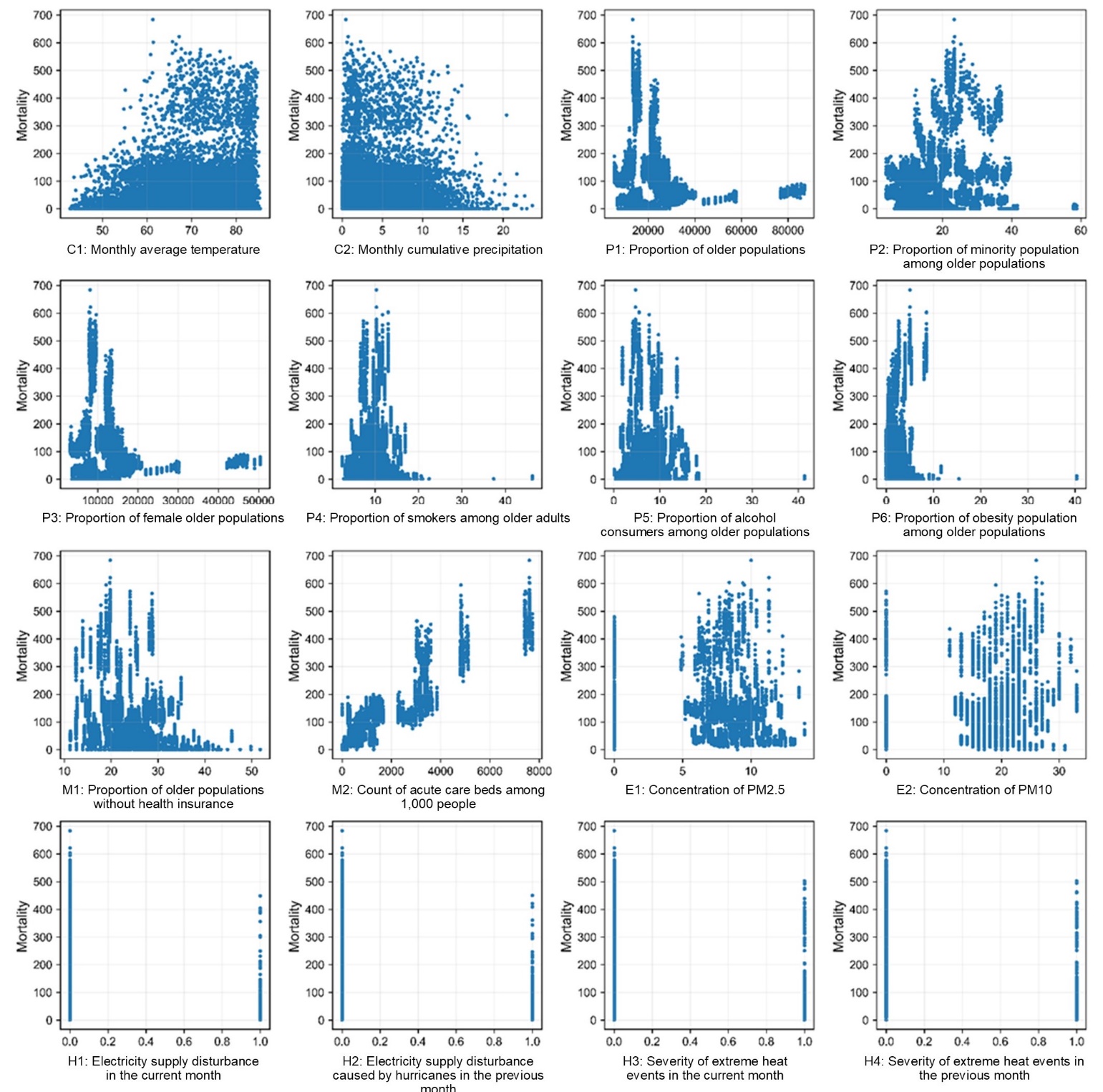


**Supplementary Figure S3** Monthly trend of explanatory variables


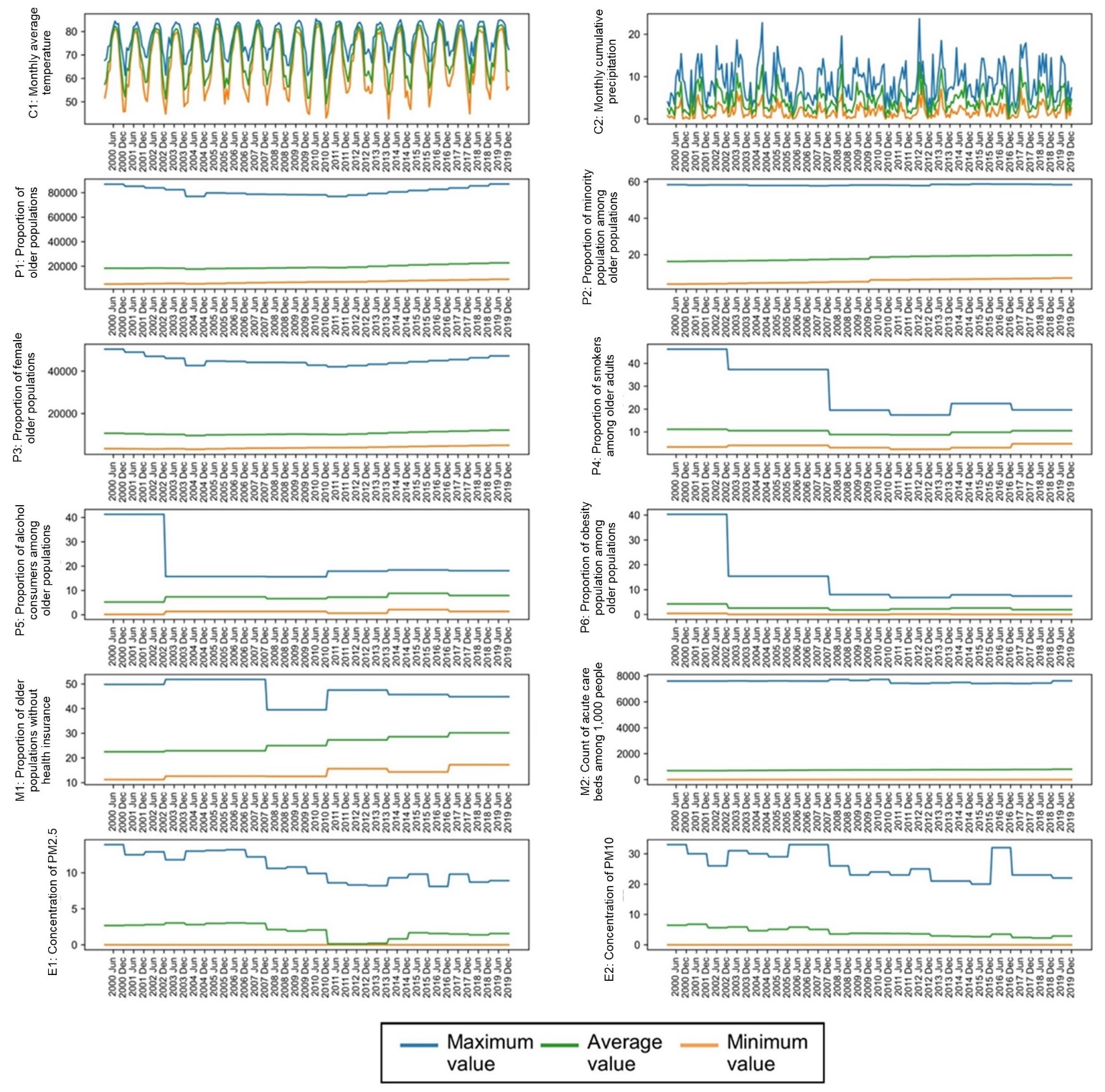


**Supplementary Table S1** List of assumptions for anticipating older adults’ mortality of heart diseases and stroke under future disaster scenarios

| Dimension of future scenarios | Assumptions |
| --- | --- |
| Climate change | **Air pollution**: Based on the projected trends of air pollution concentrations under climate change, we set the value of air pollutant concentration under RCP4.5 as zero (i.e., air pollutants can be ignored) to represent the low-emission scenarios under the future emission controls. Under RCP8.5, we set the value of air pollutant concentration as the same as in 2019, representing the business-as-usual scenarios. |
| Population growth | **Population growth under SSP2 and SSP5**: We adopted the basic assumptions of population growth under SSP2 and SSP5 in Hauer’s projections. Specifically, for high-income OECD countries (e.g., the U.S.), the population growth under SSP2 would follow the medium levels of fertility, mortality, and migration in historical records. In contrast, the population growth under SSP5 would follow the high levels of fertility and migration, while the mortality levels would be low levels based on historical records. |
|  | **Proportion of smokers, alcohol consumers, the obese population**: We regard the proportion of smokers, alcohol consumers, and the obese population in the future scenarios as the same as ones in 2019. |
|  | **Medical resource availability:** We regard the proportion of population with health insurance and the count of acute care beds among 1,000 population in the future scenarios as the same as the values in 2019. |
